# Sporadic Creutzfeldt-Jakob Disease: A Case Report and Literature Review

**DOI:** 10.7759/cureus.76589

**Published:** 2024-12-29

**Authors:** María José Sánchez Pérez, Ana S Vargas, Benito G Ceballos Vazquez Tagle, Cesar A Nieves Perez

**Affiliations:** 1 Internal Medicine, Hospital Angeles Pedregal, Mexico City, MEX

**Keywords:** cognitive and motor dysfunctions, creutzfeldt-jakob disease, neurodegenerative disease, prion desease, sporadic creutzfeldt-jakob disease

## Abstract

Prion disease is an uncommon entity characterized by exceptionally rapid neurodegenerative deterioration. There are three categories of prion disease: (1) sporadic: sporadic Creutzfeldt-Jakob disease (sCJD), sporadic fatal insomnia, and protease-sensitive prionopathy; (2) genetic: genetic Creutzfeldt-Jakob disease, familial fatal insomnia, and Gerstmann-Sträussler-Scheinker syndrome; and (3) acquired: Kuru, iatrogenic Creutzfeldt-Jakob disease, and variant Creutzfeldt-Jakob disease. Although it is an incurable disease, a specific pathophysiological mechanism exists involving neuronal loss, glial cell proliferation, absence of inflammatory response, development of vacuoles leading to a spongiform appearance, and the presence of prions. This case report describes the approach to a patient with progressive cognitive deterioration, later developing motor ataxia and difficulty in language expression. The patient was hospitalized for the diagnostic approach of autoimmune or paraneoplastic encephalitis at Hospital Ángeles del Pedregal, Mexico City, Mexico, with poor response to medical treatment and clinical worsening. Finally, a diagnosis of Creutzfeldt-Jakob disease was concluded through cerebrospinal fluid analysis. This demonstrates the diagnostic challenge this entity presents.

## Introduction

Creutzfeldt-Jakob disease is a devastating and fatal neuropsychiatric disorder characterized by a combination of cognitive and motor dysfunctions, with rapid progression marked by the aggregation of misfolded prion protein Scrapie. The disease has a global incidence of one to two cases per one million per year [1]. In our country, cases have been reported very infrequently over the past 10 years [2,3]. Although the incidence is low, the mortality rate is as high as 80%, within months of the onset of the disease, which is significant. Here, we present the case of a 73-year-old male patient who was admitted to our hospital with rapidly progressive cognitive deterioration, accompanied by ataxia and myoclonus.

## Case presentation

A 73-year-old male patient, with no known allergies and a history of well-controlled systemic hypertension, presented to the neurology clinic with difficulty concentrating, persistent confusion, bradypsychia, bradylalia, and altered visual perception for approximately three weeks. On physical examination, the patient showed no neurological focalization and had intact higher mental functions.

**Figure 1:**
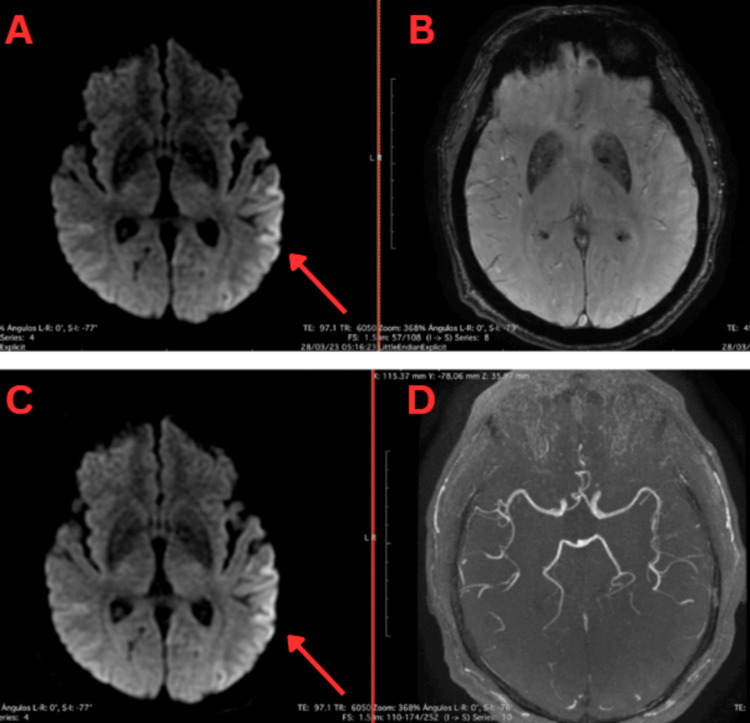
First axial brain MRI: diffusion-weighted, SWI, and MR angiography sequences Panels A and C (DWI): Diffusion sequence with apparent hyperintensity in the left posterior-parietotemporal cingulate gray matter and possibly the right temporal region, without contrast enhancement zones. Panel B (SWI) does not show significant hemorrhagic changes, and the absence of signal loss suggests no major hemorrhage. Panel D (MRA) shows intact vasculature.

An external brain magnetic resonance (Figure 1) showed cortical hyperintensity at the interhemispheric frontal level in the left-dominant parietal and temporal lobes, seen in diffusion and fluid-attenuated inversion recovery sequences. Due to these findings, the patient was admitted for further study to rule out viral meningoencephalitis, autoimmune encephalitis of paraneoplastic origin, and Creutzfeldt-Jakob disease.

Routine labs, including complete blood count, blood chemistry, and thyroid profile, revealed only grade I normocytic normochromic anemia according to WHO standards, with an unremarkable electroencephalogram for the patient’s age. Lumbar puncture revealed cerebrospinal fluid that was rock-water clear, transparent without clot, glucose of 64 mg/dL (blood glucose of 98 mg/dL), total proteins of 80.1 mg/dL, chloride of 127 mg/dL, leukocytes of 0 cells/µL, and erythrocytes of 40 cells/µL. Cerebrospinal fluid multiplex polymerase chain reaction for meningitis/encephalitis profile was negative for bacteria, viruses, and yeast, as were respective cultures. N-methyl-D-aspartate (NMDA) receptor antibody and 14-3-3 protein Creutzfeldt-Jakob tests were requested. Methylprednisolone 1 g IV boluses were initiated for five days, considering autoimmune encephalitis as the primary etiology.

A positron emission tomography scan (PET-CT) with fludeoxyglucose 18 (FDG) was performed to rule out paraneoplastic autoimmune syndrome, which ruled out a primary extra-neurological neoplastic focus, leaning the diagnosis towards non-paraneoplastic autoimmune encephalitis. Intravenous methylprednisolone was continued, and levetiracetam was initiated as seizure prophylaxis.

**Figure 2:**
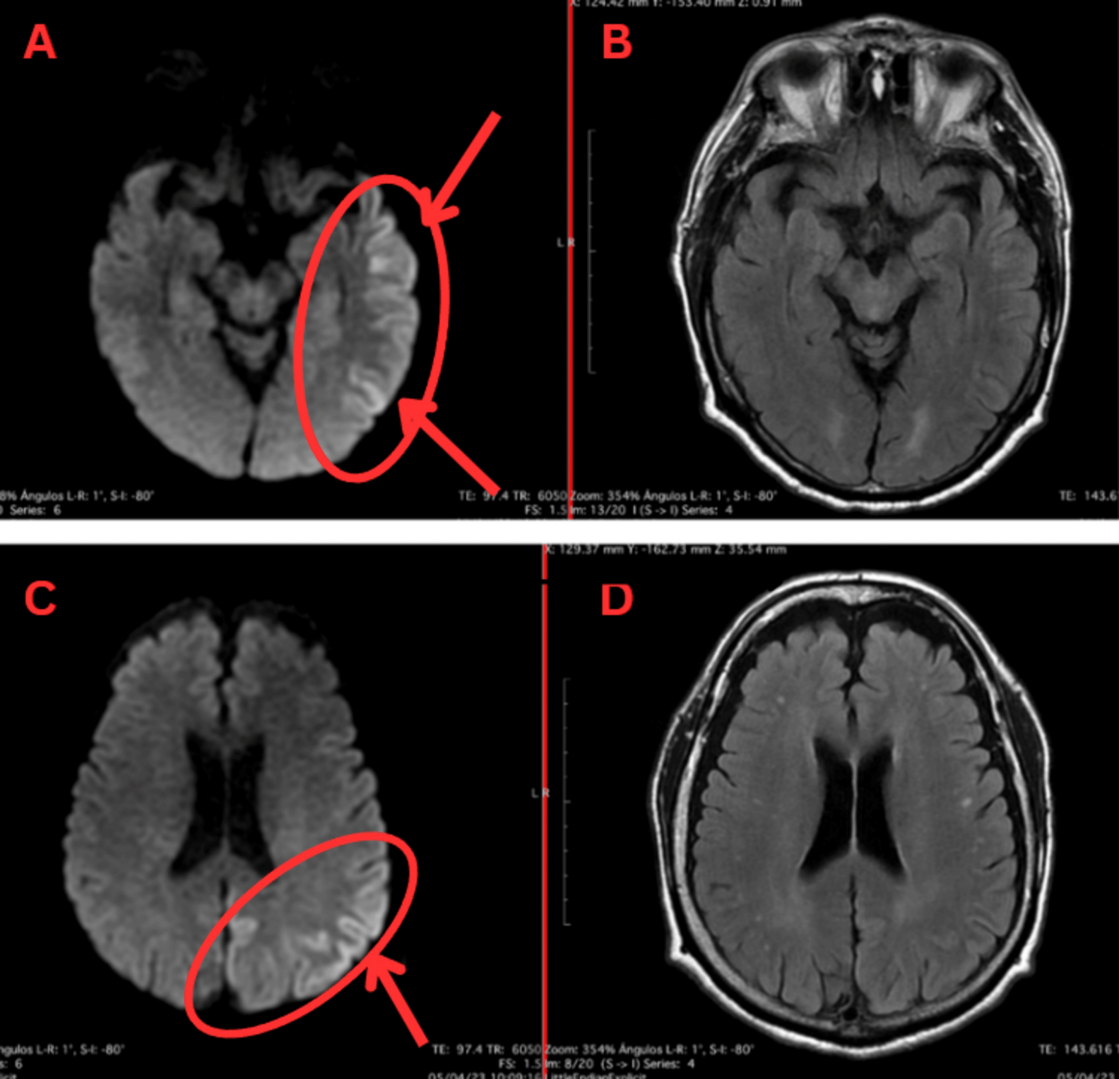
Second axial brain MRI: diffusion-weighted and fluid-attenuated inversion recovery (FLAIR) sequences Panels A and C show persistence of inflammatory changes in the left temporal and parietal cortex compared to the previous study. Panels B and D (FLAIR sequences) do not yet show significant changes.

After completing five doses of methylprednisolone boluses, the patient exhibited clinical worsening characterized by asthenia, adynamia, exacerbated bradypsychia, and difficulty in expression, leading to the suspension of levetiracetam and continuation of prednisone 75 mg daily. A cognitive test (Mexican version of MOCA) scored 69, indicating significant cognitive impairment despite treatment. A follow-up electroencephalogram showed no epileptic activity, and a control brain magnetic resonance (Figure 2) reported hyperintensities in the temporal, occipital, and parietal lobes, suggesting autoimmune versus herpetic encephalitis and persistence of inflammatory changes in the left temporal and parietal cerebral cortex better defined compared to the previous study.

Despite negative cerebrospinal fluid cultures and polymerase chain reaction, the patient continued to exhibit bradylalia and cognitive deterioration. As a result, meningeal doses of acyclovir (800 mg IV every eight hours) were initiated empirically, alongside lacosamide 300 mg/day, due to the possibility of focal disconnection seizures. In the following days, the patient experienced cognitive decline, transient motor aphasia, echolalia, and ataxic gait.

**Figure 3:**
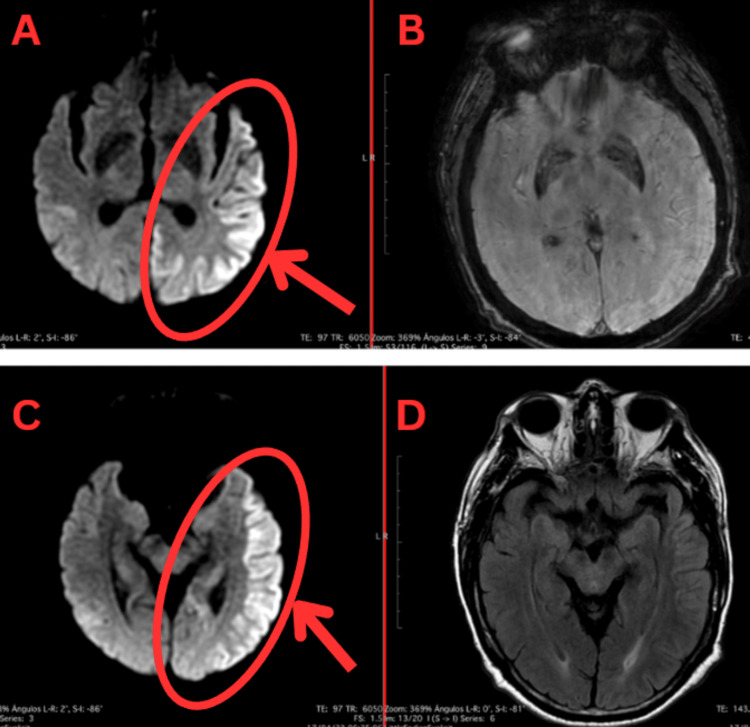
Third axial brain MRI: diffusion-weighted imaging (DWI), SWI, and fluid-attenuated inversion recovery (FLAIR) sequences Panels A and C (DWI sequences): Inflammatory changes in the left temporo-parietooccipital cortex and right parietal and temporal regions, with increased affected areas compared to the previous study. Panels B and D (SWI and FLAIR sequences) do not reveal any associated lesion.

After six days of acyclovir treatment without clinical improvement, intravenous immunoglobulin 2 g/kg distributed over five days (190 g) was initiated, and a viral hepatitis panel was requested, which was negative. Despite treatment, the patient’s cognitive decline persisted, and it was considered a case of refractory autoimmune encephalitis. A follow-up electroencephalogram remained unchanged, and a control brain magnetic resonance (Figure 3) showed persistence and extension of cortical hyperintense lesions. Treatment was escalated to rituximab 1,000 mg.

During rituximab administration, the patient experienced elevated blood pressure (160/100 mmHg) and bradycardia (38 bpm) without symptoms, leading to a temporary suspension of the medication infusion and administration of a single 5 mg dose of amlodipine, reaching 128/90 mmHg.

The rituximab infusion was then resumed without further issues, and the patient was discharged home with follow-up in an outpatient setting. Ultimately, results from the lumbar puncture for N-methyl-D-aspartate (NMDA) receptor antibodies were negative, while real-time quaking-induced conversion (RT-QUIC) of cerebrospinal fluid was positive, cerebrospinal fluid T-TAU protein was 9,753 pg/mL (reference range: 0-1,149 pg/mL), and 14-3-3 gamma cerebrospinal fluid was 37,462 AU/mL (reference range: <30-1999), meeting the Centers for Disease Control and Prevention definition and diagnosis of “probable prion disease.”

## Discussion

This neurodegenerative disease arises from hereditary disorders or sporadic mutations of the gene encoding prion protein. Types of Creutzfeldt-Jakob disease include sporadic Creutzfeldt-Jakob disease (sCJD) (the most common), familiar Creutzfeldt-Jakob disease, variant Creutzfeldt-Jakob disease, and acquired Creutzfeldt-Jakob disease.

Prion taxonomy and disease history 

Prion disease was first described in 1750 in Germany as a prion disease in sheep and goats, called scrapie. It was believed to be caused by a small filterable agent, a slow-replicating virus, as the transmissibility period was described as 22 months.

The relationship between the disease in animals and humans is described, and the endemic neurodegenerative disorder in cannibalistic tribes of Papua New Guinea called Kuru was identified. Cerebral vacuolization in the absence of an inflammatory response and the similarities between Kuru patients and animals with scrapie disease have been described [4].

in 1982, an infectious agent was purified, which is known to be the cause of the disease, proposing the name "prion" 2, derived from "proteinaceous infectious particle" to distinguish it from viruses due to its lack of deoxyribonucleic acid (DNA). In 1922, the name "Creutzfeldt-Jakob" was given as German doctors Creutzfeldt and Jakob previously described the clinical picture of spasticity and rapidly progressive neurological degeneration in their patients, though it was first described as "Creutzfeldt-Jakob disease" by Walther-Spielmeyer [4].

Pathophysiology

The function of prions is not well established; however, it is known that prion protein, a glycoprotein expressed in the brain, is transcribed from a chromosomal gene (PRNP) located at locus 20p13. Normally, prion protein (referred to as PRPc, “c” for cellular) is a globular protein composed of three alpha-helical segments and two short beta strands forming a beta-sheet.

This normal prion protein is modified to a pathological form by the addition of a glycosylphosphatidylinositol residue that anchors the carboxyl-terminal end of the mature protein to the outer surface of the cell membrane, converting it into a prion protein with abnormal conformation (called PRPsc, “sc” for scrapie), which has an extensive beta-sheet structure not present in PRPc.

The accumulation of PRPsc causes neighboring PRPc proteins to mimic the abnormal conformation, acquiring the PRPsc form. The mechanism by which PRPc proteins adopt the abnormal PRPsc form in sCJD is unknown. However, the accumulation of prion proteins with abnormal PRPsc conformation in neurons leads to neuronal death, resulting in vacuolated spaces in the cortex, giving the brain a spongiform appearance.

Epidemiology

Creutzfeldt-Jakob disease is the most common prion disease, though it remains a rare condition. The vast majority of cases are sporadic (85-95%), with 5-15% being genetically caused; iatrogenic and variant forms account for less than 1% of cases. Approximately one to two cases of sCJD occur per 1,000,000 individuals globally each year [1].

The average age of onset for sCJD is 62 years, though cases have been reported in individuals over 80 years old and in younger adults. Those with variant Creutzfeldt-Jakob disease and iatrogenic Creutzfeldt-Jakob disease tend to be much younger. Conversely, individuals with genetic Creutzfeldt-Jakob disease tend to be slightly younger than those with sCJD. There is no sex preference for the disease [5].

The incidence of the disease varies geographically. It tends to be 30-100 times higher in North Africa, Israel, Italy, and Slovakia, primarily due to genetic causes. In Mexico, a series of 24 cases of sCJD was reported, with 75% being female. The mean age of disease onset was 59.29 ± 6.54 months. Seven cases showed total positivity for Tau, five were positive for protein 14-3-3, and three were positive for phosphorylated tau in cerebrospinal fluid analysis [2].

The genetic risk factor for sCJD is the prion protein (PRNP) gene, particularly codons 129 and 219, which provide protection when heterozygous and increase the risk when homozygous. However, no risk factors for the development of the disease have been established to date.

Clinical presentation

Signs and symptoms of Creutzfeldt-Jakob disease tend to be heterogeneous; however, the disease is characterized by rapid neuropsychiatric deterioration, with death typically occurring within one year of symptom onset. The disease manifests with prodromal, neurological, and neuropsychiatric symptoms. Prodromal symptoms are variable and may be subtle, including sleep disturbances, particularly hypersomnia, headaches, and nonspecific fatigue [6].

Neurological symptoms primarily manifest as cerebellar abnormalities, such as nystagmus and ataxia, as well as extrapyramidal symptoms, including hypokinesia, bradykinesia, dystonia, and rigidity. Additionally, corticospinal tract abnormalities are observed, such as hyperreflexia and extensor plantar responses (Babinski's sign), along with spasticity. Patients experience myoclonus, particularly when startled, although these can occur at any time during the course of the disease [7].

Neuropsychiatric symptoms are characterized mainly by rapidly progressive dementia, behavioral changes, and deficits affecting higher cortical functions, such as aphasia, apraxia, and frontal lobe syndromes. Initial manifestations include difficulties with concentration, judgment, and memory. Mood changes, such as apathy and depression, are quite common, while euphoria, emotional lability, and anxiety are less frequent [8]. Most patients rapidly decline to a state of akinetic mutism, with 80% of affected individuals dying within the first year.

Diagnosis

In the 1970s, the diagnosis was made through biopsy, where characteristic findings included neuronal loss, reactive gliosis, vacuolated spaces with a spongiform appearance, and the absence of inflammatory signs.

In the 1980s, electroencephalogram (EEG) became part of the diagnostic approach, showing a classic pattern described as a slow background rhythm interrupted by biphasic or triphasic sharp wave complexes, either bilateral, periodic, or synchronous.

By the 1990s, quantification of protein 14-3-3 was described. These regulatory proteins bind to peptides involved in cellular transduction and growth, with a sensitivity of 92% and variable specificity, averaging 80%, and 90% in typical clinical cases [9].

Around the 2000s, magnetic resonance imaging was employed, with a primary focus on T2-weighted sequences, diffusion sequences, and fluid-attenuated inversion recovery (FLAIR) sequences, showing characteristic findings of spongiform degeneration, hyperintensity in the cortex described as the “ribbon sign,” and hyperintensity in the basal ganglia, particularly the anterior putamen. The combination of the latter two radiographic findings provides a sensitivity of 67% and specificity of 93% [9].

Genetic testing with evidence of PRNP gene mutation is available but is not very sensitive, as 85% of patients present with the sporadic type of Creutzfeldt-Jakob disease without genetic mutations [9].

Since 2010, the real-time quaking-induced conversion (RT-QuiC) test has been established for diagnosis, which detects in vitro aggregation of recombinant PrP, with a sensitivity of 90% and specificity of 99%. This test is the most commonly used to date. Research into the diagnostic utility of serum PrP levels continues [9].

Treatment and prognosis

Creutzfeldt-Jakob disease is a fatal condition characterized by rapid progression toward disability, significantly impacting patients and their caregivers [10]. Approximately 90% of patients die about one year after diagnosis (4.4-14 months) from the onset of symptoms. Diagnosis usually occurs at two-thirds of the disease course, leaving very little time for patients and families to prepare for the end stage of life. Due to the lack of effective treatment, healthcare providers focus on managing and promoting a good quality of life through neuro-palliative measures [10,11]. Treatment is complex and requires multidisciplinary management involving neurology units, hospices, home care, and hospital care. An early approach by the medical team is essential for prompt intervention regarding diagnosis and prognosis, as the disease is relatively unfamiliar. Early intervention from psychological and psychiatric services for both patients and families is advisable. Given the high diversity and complexity of physical symptoms, experienced palliative care physicians are necessary [12,13].

Table 1 presents the common symptoms in patients with Creutzfeldt-Jakob disease and their corresponding treatments.

**Table 1:** Medical management of common symptoms in the end stage of Creutzfeldt-Jakob disease Source: Refs. [12,13]

Symptom	Treatment
Myoclonus	Minimal movements when touching, turning, or repositioning the patient
	Maintain a calm environment
	Levetiracetam
	Valproate
	benzodiazepines like clonazepam
Spasticity	Although less effective in the presence of muscle rigidity, responds to medications, such as:
	Dantrolene
	Diazepam
Ataxia	In most cases, it is extremely difficult to treat, and, in many cases, there are no effective medications available
Visual hallucinations	Respond well to medications used in Alzheimer’s disease, such as:
	Donepezil
	Galantamine
	Rivastigmine
Aggression and agitation	Atypical antipsychotic medications like: quetiapine
	Risperidone
	Olanzapine
	In some cases, diazepam can also help
Pain	Pain assessment is difficult due to communication problems
	The potential for pain exists in the presence of spasticity, hyperreflexia, bladder, and bowel infections
Paresthesia and/or dysesthesia	Gabapentin
	Pregabalin
	Amitriptyline
Exaggerated startle reflex	Non-pharmacological approaches like calmly talking, using gentle and minimal touches, ensuring minimal sound, playing soft music, and using dim lighting
Difficulties swallowing (dysphagia)	Thickened fluids and pureed foods
	Proper positioning to reduce the risk of aspiration
	Any decision to introduce food through a nasogastric tube or feeding via a gastrostomy should be carefully considered and negotiated with the family
Increased saliva due to difficulty swallowing	Can be managed with medications that reduce saliva, such as atropine drops or hyoscine patches
Oral infections (e.g., candidiasis)	Oral care is important to prevent infections and maintain comfort
	It can be challenging in the presence of myoclonic jerks
	The administration of analgesics like paracetamol may help facilitate oral care
Urinary frequency	Urinary antispasmodics like oxybutynin
	Tolterodine
Urinary incontinence	Frequent visits to the bathroom
	Using protectors for incontinence
	If there is concern about skin ulcers or nearing the end of life, urinary catheterization may reduce the need to touch and frequently turn the patient while changing the bed protectors
Constipation	Often due to fluid intake, immobility, and neurological effects on the intestine
	The use of laxatives can help manage symptoms of stool retention and anxiety related to constipation

Current studies are exploring potential functional therapies, but none have yet shown improvement in symptom development, severity, or prognosis. Some of these treatments include flupirtine, quinacrine, doxycycline, pentosan polysulfate, and genetic therapies, such as anti-PRN 100 antibodies [11-13].

## Conclusions

Creutzfeldt-Jakob disease represents a rare pathological entity, characterized by relatively rapid progression and variable clinical manifestations, which complicates early diagnosis and results in an unfavorable prognosis. Due to its low prevalence and the intricate nature of the disease, there is currently no specific treatment available, limiting interventions to symptomatic approaches aimed at improving the patient's quality of life. This case underscores the rarity and diagnostic challenges of the disease, including limited access to laboratory tests, and highlights the need for timely and accurate diagnostic efforts to avoid inappropriate treatments in cases of uncertain diagnosis. Future efforts should focus on enhancing early recognition and diagnostic accuracy to improve the management of this fatal condition.
